# SimpleITK Image-Analysis Notebooks: a Collaborative Environment for Education and Reproducible Research

**DOI:** 10.1007/s10278-017-0037-8

**Published:** 2017-11-27

**Authors:** Ziv Yaniv, Bradley C. Lowekamp, Hans J. Johnson, Richard Beare

**Affiliations:** 1 TAJ Technologies Inc., Bloomington, MN 55425 USA; 2 National Library of Medicine, National Institutes of Health, Bethesda, MD 20894 USA; 3 MSC LLC, Rockville, MD 20852 USA; 4 Department of Electrical and Computer Engineering, The University of Iowa, Iowa City, Iowa 52242 USA; 5 Department of Medicine, Monash University, Melbourne, VIC 3168 Australia

**Keywords:** Image analysis, Open-source software, Registration, Segmentation, R, Python

## Abstract

Modern scientific endeavors increasingly require team collaborations to construct and interpret complex computational workflows. This work describes an image-analysis environment that supports the use of computational tools that facilitate reproducible research and support scientists with varying levels of software development skills. The Jupyter notebook web application is the basis of an environment that enables flexible, well-documented, and reproducible workflows via literate programming. Image-analysis software development is made accessible to scientists with varying levels of programming experience via the use of the SimpleITK toolkit, a simplified interface to the Insight Segmentation and Registration Toolkit. Additional features of the development environment include user friendly data sharing using online data repositories and a testing framework that facilitates code maintenance. SimpleITK provides a large number of examples illustrating educational and research-oriented image analysis workflows for free download from GitHub under an Apache 2.0 license: github.com/InsightSoftwareConsortium/SimpleITK-Notebooks.

## Introduction

Research and educational activities that include analysis of biomedical images are carried out by diverse communities that include physicians, biologists, biomedical engineers, physicists, mathematicians, computer scientists, and others. As a consequence, similar image analysis tasks are performed by people with significantly different levels of software development skills. Less-experienced software developers often use programs that facilitate high-level computational workflows via graphical user interfaces such as 3D Slicer [8] and Fiji [24]. More experienced software developers often prefer to directly use computational toolkits such as the Insight Segmentation and Registration Toolkit (ITK) [13]. Many in this diverse community of scientists have limited formal training and skills as programmers or software developers. This observation was described in [10]: “In the Future, Everyone Will Be a Programmer for 15 Minutes”. To support their work, these non-expert developers need tools to address the challenges of testing and maintaining their software creations.

An essential requirement for confirming any conclusions described in a scientific publication, including biomedical image analysis findings, is reproducibility [7, 21]. Satisfying this requirement is challenging when the computational workflow is manual, which is often the case when using programs with graphical user interfaces. In these situations, the workflow is not documented in an automated manner. The need for documented computational workflows is likely the reason why both 3D Slicer and Fiji offer automated scripting capabilities. While this addresses the reproducibility issue, in many cases, the flexibility of such solutions is limited when compared to directly using an image analysis toolkit such as ITK. Conversely, the use of ITK requires considerable proficiency in the C**++** object-oriented programming language, a relatively specialized skill.

Another significant aspect of modern scientific research is its collaborative nature. Collaborations between multiple groups at different institutions and possibly countries is common and appears to be a hallmark of the current scientific landscape [1, 2]. The work presented here is one such example. Developing biomedical image analysis workflows in a distributed collaborative setting requires sharing of data, computational models in natural language including their mathematical derivations, source code, and the current results both numeric and in graphical form. For active collaborations, all four components need to be synchronized so that participants in the research endeavor are aware of the current state of affairs. In most cases, the four components are separate: (1) there is a document describing the model and mathematical derivation, (2) there are source code files, ideally, managed using a version control system, (3) with both originating data, and (4) summary result files. The management of the separate components is not an ideal approach to communication between participants because it requires that each participant develop connections between sections in the model description, the implementation, and the corresponding results. These issues were clearly identified in the 1980s, with the proposed solution being literate programming [16]. In the literate programming approach, the computation description using natural language, figures, and accompanying mathematical derivations are embedded with the implementation in a single document. This approach facilitates improved understanding in the educational setting and can enhance collaborative work. Unfortunately, while the idea is attractive, it was not widely adopted till rather recently.

In this work, we present an image-analysis environment which addresses the challenges described above. We enable flexible reproducible computational workflows using a simplified interface to ITK that is accessible to programmers with varying levels of experience. The workflows are implemented using a web-based literate programming environment that provides opportunities for improving collaborations. Finally, all necessary components are combined in a testable, maintainable image analysis environment with transparent data sharing via online repositories.

## Materials and Methods

Three elements that comprise the image-analysis environment are the SimpleITK toolkit, the SimpleITK Jupyter notebooks, and the development infrastructure.

### SimpleITK

#### Overview

SimpleITK, originally introduced in [18], is a simplified programming interface to the algorithms and data structures of the Insight Segmentation and Registration Toolkit. SimpleITK has specializations for multiple programming languages including C+ +, Python, R, Java, C#, Lua, Ruby, and TCL. The SimpleITK interfaces address the primary challenge associated with using ITK algorithms by directly allowing scientific domain experts to use the most common forms of these algorithms in any one of the programming languages that they are familiar with. SimpleITK enables users to use the skills they have rather than requiring a significant amount of effort to become proficient in C+ +.

Three characteristics of ITK that make it a phenomenal platform for efficiently implementing complex medical image analysis tools also require advanced software development experience. The first characteristic is that the toolkit is distributed in source code form, requiring the developer to compile ITK before they can use it. The second characteristic derives from ITK’s extensive use of the C+ +template mechanism to provide data-type flexibility. Finally, ITK components are designed for use as part of a data analysis pipeline. ITK elements are connected sequentially to construct the pipeline with no computations performed during pipeline construction. This powerful pipeline architecture adds complexity that is a significant barrier to implementation for many ITK newcomers. Each of these power-enabling characteristics requires specialized understanding of advanced programming concepts.

SimpleITK shortens the time it takes to access the ITK toolkit algorithms by trading-off potential flexibility and performance for global ease of use. There are binary distributions for a variety of programming languages. It abandons the use of templates in favor of a small subset of a specific pixel, image, and filter types which correspond to the most widely used data-type combinations. And finally, it hides the use of the filter pipeline in favor of the more simple programming approach whereby the user provides input to a filter which immediately returns the output.

When introduced in [18], SimpleITK included a large number of components for image filtering and segmentation. Since then, the toolkit has been significantly extended to include over 280 ITK filters, with the primary addition being the incorporation of the ITK version 4 (ITKv4) registration framework [3]. This addition completes the SimpleITK functionality, with comprehensive support for image filtering, segmentation, and registration. We next describe the new registration framework, starting with its two principal data elements, transformations, and images.

#### Transformations

The toolkit supports all of the transformation types supported by ITK, both those with a global domain such as rigid transformations and those with a local domain such as displacement fields. In addition to the standard transformations, with the introduction of the ITKv4 registration framework, we include its associated composite transformation.

**Fig. 1:**
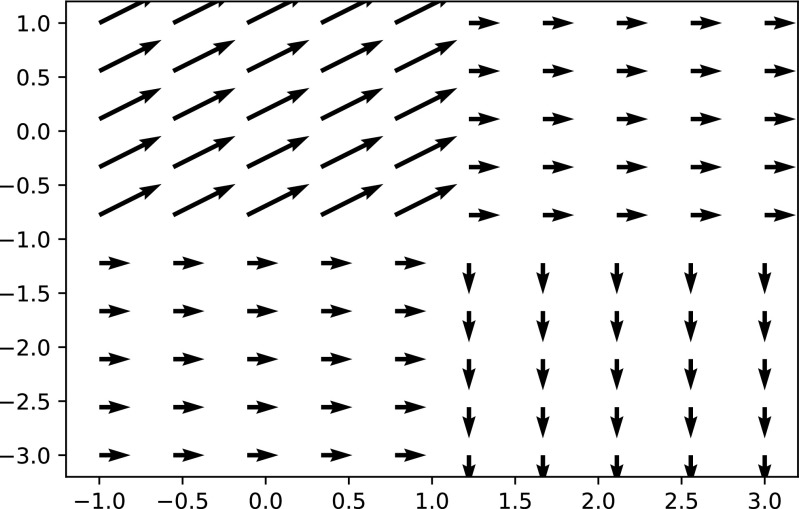
Visualization of a single composite transform representing a translation (*x* = 1,*y* = 0)and two deformation fields. The first is a uniform translation (*x* = 1,*y* = 1)for all locations in the domain [− 1 − 1] × [1,1]. The second is a uniform translation (*x* = − 1,*y* = − 1)for all locations in the domain [1,− 3] × [3,1]. Note that the arrows are scaled for improved visualization

The composite transform allows representation of multiple transformations applied one after the other. Each of the transformations is added into the composite transform using stack-based semantics, first added last applied. For instance, to represent *T*_*comp*_ = *T*_*affine*_(*T*_*rigid*_(*x*)), we first add *T*_*affine*_ to the composite transform and then *T*_*rigid*_. The composite transform enables easy representation of a transformation with a global domain that also includes multiple transformations with local domains in a single transformation. This is a useful feature for representing a complex transformation with multiple deforming regions while other regions are only effected by the global transformation. Figure 1 illustrates this scenario. In the context of registration, the use of a composite transformation requires some attention. The parameters that are optimized are only those of the last added transformation and not all of the parameters which comprise the complete transformation. That is, while *T*_*comp*_above is an affine transformation, registration will only modify the parameters of *T*_*rigid*_ during the optimization process.

#### Images

The second principal data element in SimpleITK are images. To facilitate adoption of SimpleITK in various languages, indexing conventions and access to sub-regions of the image, often referred to as *slicing*, are language specific. For example, in the R language indexes start at one, while in the Python language they start at zero. Thus for the same 2 × 2 image, valid index values in Python are in [0,1]while in R they are in [1,2].

**Fig. 2:**
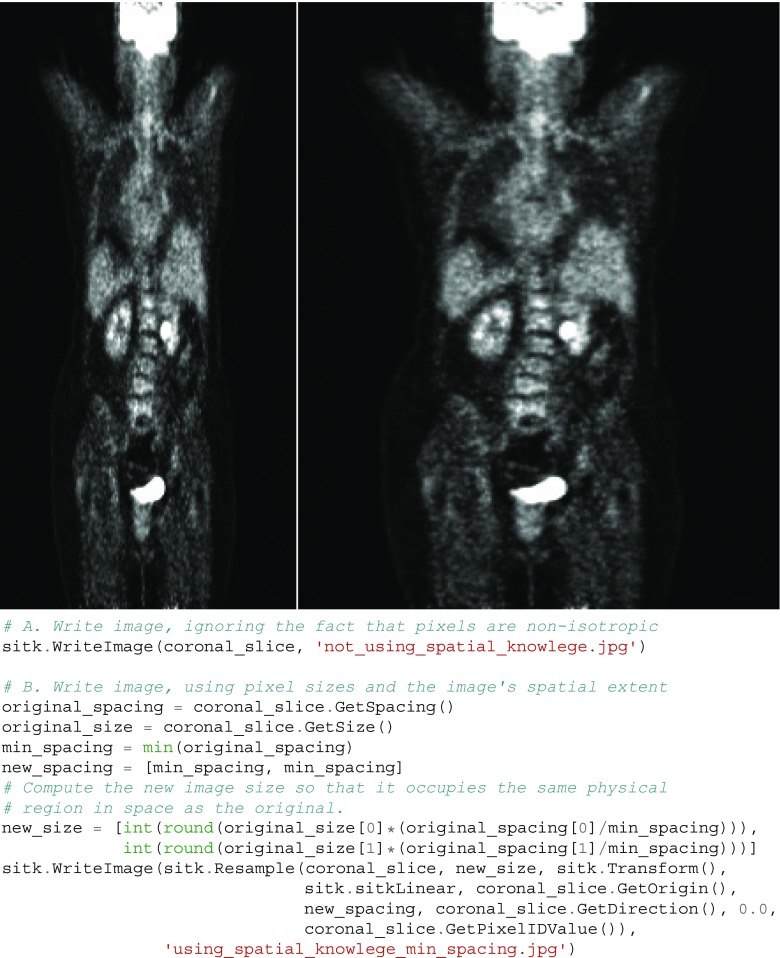
Coronal slice extracted from PET volume: (left) original intensity values saved in jpg format, which does not take into account the physical spacing between sample (right) intensity values isotropically resampled and then saved in jpg format. To resample the original intensities onto an isotropic grid, we use the image’s spatial information. The resampled image should occupy the same physical region in space as the original. The code above illustrates that resampling takes this into account by specifying that both images have the same origin and axes direction but different pixel spacings (4.0 × 2.5 vs. 2.5 × 2.5 mm) and consequentially grid sizes (168 × 344 vs. 273 × 344)

A fundamental tenet of ITK and consequentially of SimpleITK is that *images occupy a physical region in space*. This tenet is critical for adoption of tools to real-world medical imaging applications. An image is thus defined by its voxel level content and additional meta-data including its origin, the spacing between pixels and a direction cosine matrix defining the physical direction of each of the image axes. This differs from most image-analysis libraries that treat images as simple arrays of values. Figure 2 illustrates the use of spatial information required to correctly resample an image. Not to preclude the use of external image-analysis libraries, SimpleITK provides functionality for transferring the image data to and from array structures in the native language.

#### Registration

SimpleITK provides an easily configurable multi resolution registration framework supporting a variety of transformation types, optimization algorithms (both derivative free and derivative based), similarity metrics, and interpolators. The framework uses a ImageRegistrationMethod component to represent a registration algorithm and the user configures the underlying ITKv4 components via this component. In addition, some variations of the Demons registration algorithms are implemented independently from the ImageRegistrationMethod as they do not fit into the framework given that they are limited to computation of a deformation field and assume the intensity values of corresponding structures do not change.

A novel aspect of the ITKv4 registration framework which often confuses newcomers is the use of a virtual image domain. This feature is also included in SimpleITK, and involves three transformations instead of the single transformation used by the traditional registration approach. The default values of the registration framework allow one to easily ignore this complexity reverting to the use of the traditional domains where a single transformation maps between the fixed image domain and the moving image domain. The motivation for introducing the virtual image domain is that it results in a symmetric registration process where both images are treated similarly with intensity values acquired via interpolation at off grid locations in both images. The registration interface allows the user to set three transformations:

- *T*_*f*_: maps points from the virtual image domain to the fixed image domain, never modified.
- *T*_*m*_: maps points from the virtual image domain to the moving image domain, never modified.
- *T*_*opt*_: composed with *T*_*m*_, maps points from the virtual image domain to the moving image domain, modified during optimization.

Using these transformations, a point in the fixed image domain is mapped to a point in the moving image domain as follows:

$${~}^{m}\mathbf{p} = T_{opt}\left( T_{m}\left( T_{f}^{-1}(^{f}\mathbf{p})\right)\right) $$

By default the virtual and fixed image domains coincide, *T*_*f*_ = *I*. To use the framework in the traditional manner, the user need only provide an initial value for *T*_*opt*_ which is modified during the registration process.

An additional constraint imposed by the ITKv4 registration framework is that the pixel type of registered images is required to be a floating point type. In SimpleITK, these are the sitkFloat32 and sitkFloat64 types. This constraint is only a minor inconvenience, as the user can either enforce this when the image is read from disk by specifying the reader’s output pixel type or they can cast the pixel type to a float type prior to registration using SimpleITK’s Cast function.

**Fig. 3:**
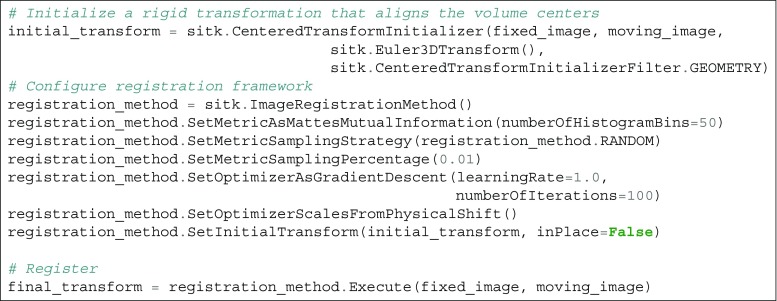
SimpleITK Python code for multi-modality rigid registration. Initialization aligns the geometric centers of the two volumes. The optimized metric is mutual information using 1% of the image voxels and a gradient descent optimizer. A novel feature of the ITKv4 registration framework is the option for automatic scaling of the optimized parameters so that a unit change in each parameter will have similar effects in physical space, the SetOptimizerScalesFromPhysicalShift function. This is of particular importance with rigid registration where our parameter space consists of rotation angles in radians and translation in millimeters

A code listing illustrating the use of SimpleITK for multi-modal rigid registration is shown in Fig. 3.

### SimpleITK Jupyter Notebooks

**Fig. 4:**
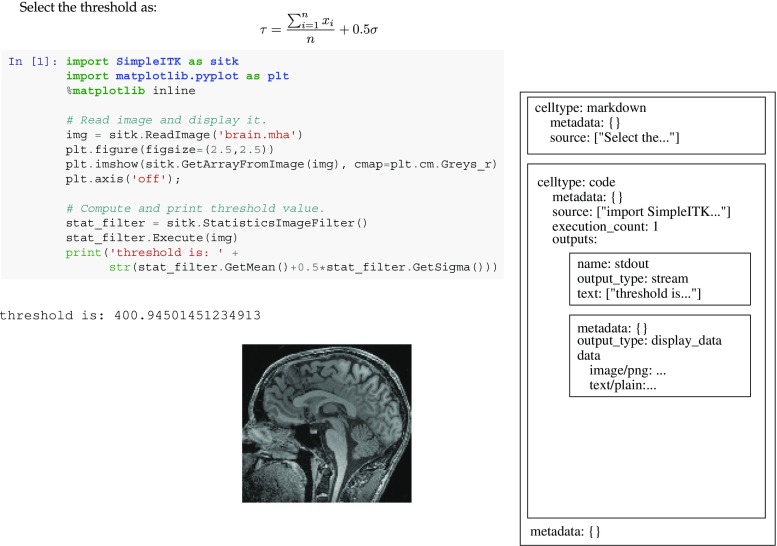
Minimal representation of a SimpleITK Jupyter notebook comprised of a markdown cell and code cell with output. On the left is the rendered notebook and on the right is its JSON representation. Note that all cells and the notebook include a meta-data object. This allows developers to store additional information in the notebook which is ignored when rendered by the Jupyter application. In SimpleITK notebooks, this meta-information is used to facilitate notebook testing

Originally introduced in 2011 as IPython notebooks, Jupyter notebooks (www.jupyter.org) are an open-source interactive web-based application facilitating literate programing [15, 25]. Jupyter notebooks support a large number of programming languages via language backends, referred to as Jupyter kernels. The kernels communicate with the system using Jupyter’s communication protocol. A notebook is a JSON-based document which is analyzed and displayed by the Jupyter application. A short SimpleITK notebook and its JSON structure are shown in Fig. 4.

SimpleITK Jupyter notebooks are available in two programming languages, Python and R. A SimpleITK notebook is a single document containing the description of an image-analysis workflow using equations and text, the executable implementation of the workflow, and figures and tables generated by running the code. The code is divided into logical sections often referred to as “code chunks” that are executed interactively, providing a natural setting for exploring the effect of parameter values on algorithm performance. This form of exploration can be done either by modifying the code chunk and re-running it, or by using graphical user interface components that are also part of the document. Another immediate benefit of executing individual code chunks is that one can choose to run certain chunks only once while others can be repeatedly executed. For example, users will run a data loading chunk once while an algorithmic chunk is run multiple times exploring a variety of approaches to solving the same analysis task.

#### Resource Control

A desirable feature of an exploratory data analysis environment is that it readily facilitate quick turnarounds. That is, one should be able to quickly explore the effects of various algorithmic selections. A straightforward approach to facilitating quick turnarounds when working on image-analysis workflows is simply to use smaller images. The use of sub-sampled images as the input and not the original full resolution data speeds up the development process and enables one to quickly experiment and evaluate various options. Additionally, in some cases, the reduced memory footprint may be a requirement due to system memory constraints. In our case, we are using the CircleCI (www.circleci.com) continuous integration service whose virtual-machines only provide 4Gb of RAM.

Ideally, switching between the use of full-resolution and sub-sampled data should not require code modification and is nearly transparent to the developer. To optionally enable this functionality, we use an environment variable in combination with a script that is run at the beginning of the notebook. When the environment variable is set, the standard image reading functionality is replaced by reading followed by resampling. The automated resampling is implemented using the decorator design pattern [9]. SimpleITK’s original ReadImage function is replaced by a decorated version which performs resampling. From the developer’s standpoint, this is transparent with images always loaded using the ReadImage function. Whether these are full-resolution images or sub-sampled ones depends on the environment variable setting.

#### Image Display and Interaction

As the goal of the SimpleITK image-analysis notebook environment is to serve as an exploratory setting for developing image-analysis workflows, we also need to address the requirement for image display and interaction. SimpleITK itself does not provide visualization components though it does facilitate image viewing using external programs. The toolkit includes a Show command which by default will write the given image to a temporary directory and attempt to launch the ImageJ/Fiji [24] viewer to display it. Other viewers such as 3D Slicer and ITK-SNAP can also be used in place of ImageJ by setting an environment variable.

The use of an external viewer has several limitations. First, the visualization is external to the notebook and is not shared with collaborators when sharing the notebook. Second, this approach can only visualize static results, and we would also like to animate algorithm progress (e.g., similarity metric values as registration progresses). Finally, and most importantly, it does not allow the user to easily interact with the image to generate input for the following phases of the analysis workflow (e.g., localizing seed points for segmentation).

The solution we provide for these challenges is to use native language facilities supported by the R and Python kernels to construct appropriate visualization components. For the R notebooks, we utilize the ggplot2 package [28] and for the Python notebooks, we utilize the matplotlib [11] and ipywidgets packages. The support for inline visualization and interaction for the R kernel is currently limited, and therefor the R-based notebooks are still limited to displaying static inline visualizations.

In the Python-based notebooks, we provide a dynamic display of registration progress using SimpleITK’s callback mechanism to record registration progress data combined with custom plotting functions. In the general case, we provide functions which display the similarity metric value throughout the registration process. When additional information is available such as corresponding target points in the two image domains we also provide functions for displaying the target registration error alongside the similarity measure value. This form of visualization allows the developer to gain insight into the appropriateness of a specific similarity measure. When the similarity metric is appropriate for the registration task, we expect to see a decrease in the similarity measure corresponding to a decrease in the target registration error.

We provide several components for display and interaction purposes that are useful for obtaining input for various registration and segmentation tasks: Registration PointDataAquisition, PointDataAquisition, MultiImageDisplay, and ROIDataAquisition. All components allow the user to zoom and pan the displayed images as part of the supported interactions.

The RegistrationPointDataAquisition component allows one to acquire point data in two volumetric image domains either as input for paired point registration or for the acquisition of corresponding point pairs for registration evaluation. The component operates in two modes, one for data acquisition and the other for visual inspection of registration results. In data acquisition mode, the two volumes are displayed side by side and the user can localize corresponding points. Undoing the last point localization and clearing all data are supported. The interface forces the user to localize a point in each volume interleaving between the two. In visual inspection mode, in addition to the images, the user is required to provide a transformation that maps between the two volumes. In this mode, when the user localizes a point in one volume, it is mapped and displayed on the other volume using the given transformation or its inverse; this is sometimes referred to as *linked cursor mode*.

The PointDataAquisition component allows one to display a volumetric image and localize points as input for various segmentation algorithms that require user-supplied seed points. The component also allows one to add pre-determined point indexes with the constraint that they are inside the image index bounds. Note that the output from this component are the physical coordinates of the points in the image’s coordinate system and not the point indexes.

The MultiImageDisplay component allows us to display multiple volumetric images with the same dimensions side by side using a single slider to scroll through the image stack. This is a useful interface for qualitative evaluation of image registration and segmentation results. In the registration case, prior to using the interface one image is resampled to the grid of the other so that they share the spatial extent and dimensions. In the segmentation case, the results of several segmentation algorithms or segmentation steps can be visually compared side by side.

Finally, the ROIDataAquisition component displays a volumetric image and allows us to interactively select one or more box-shaped regions of interest. This is useful for creating masks for registration, limiting the computation of the similarity measure to the regions of interest or for segmentation purposes such as determining a local threshold in a region of interest.

### Development Infrastructure

A key aspect of a sustainable and maintainable development environment is the infrastructure and associated development process. Our development process follows best software engineering practices including code reviews, the use of a version control system, and incorporation of continuous integration testing so that the status of the current code and any code under future consideration is available online.

We use the git version control system and the GitHub (www.github.com) service to distribute the SimpleITK Notebook environment. We therefore follow a GitHub centric workflow. In our case, the authoritative repository is found under the Insight Software Consortium organization. No developer directly commits changes to this repository. Instead, core and external developers fork create a copy of the authoritative repository on GitHub. All new code is developed using the topic branch-based git workflow approach [4]. Once a topic branch is uploaded to a forked repository, the developer issues a pull request to the main repository. The pull request automatically triggers testing using the CircleCI continuous integration testing service. Concurrently, the code is reviewed by the team. Once the code is approved by the team and passes the testing, it is merged into the master branch of the authoritative repository.

#### Testing

To test the notebooks, we provide a testing script which is distributed as part of the SimpleITK notebook environment. In general, a notebook is comprised of three types of elements: (1) markdown cells containing natural language, markdown elements, figures and links to online resources; (2) code chunks that are executed; and (3) output from the code chunks. Testing includes a static analysis of the notebook addressing each of these elements and a dynamic analysis which only deals with executing the code cells.

In the static notebook analysis, we start with the markdown cells. The script checks spelling using a US English dictionary with an additional set of exceptions that include various acronyms and non-standard words such as “SimpleITK.” In addition, the validity of all external URL links is tested to ensure that the links are not stale. We then analyze the code cells, spell checking all of the inline comments and checking for the existence of output from the code.

The output from code chunks presents a unique problem from a version control standpoint. Minor changes to output are common for many algorithms that have a stochastic component (i.e., registration). Thus, the same code will produce slightly different output every time it is run. If the output is included as part of the notebook, from the version control viewpoint, these are changes just like any other. Viewing these changes as equivalent to changes in code and textual content has the potential to result in many superfluous commits and a history of changes that for all intents and purposes should be ignored. We solve this issue by having the testing framework check that notebooks do not contain any output. Attempting to submit a notebook containing output is considered an error and the pull request is rejected.

In the dynamic notebook analysis, we use the Jupyter nbconvert tool. This tool allows the testing script to execute the notebook and convert it to a variety of formats. In our case, we use the same JSON Jupyter notebook format as the input with the output written to a temporary file. A key feature of our notebooks is that they include code that will produce errors. The purpose of this code is to illustrate common usage mistakes and their associated errors. When running a notebook, the default behavior is for execution to stop once an error is encountered. We use the nbconvert tool option which indicates that execution should continue even after an error is encountered. Once the notebook execution is completed, we analyze all of the outputs.

Each code cell has associated JSON meta-data information that is not displayed by the Jupyter application. We use this meta-data to mark code cells that are expected to generate an error and code cells that potentially generate one. Each entry also identifies the expected error message. The former type of entry is used to identify intentional errors included in the code. These errors illustrate expected behavior such as adding the intensity values of two images that do not physically overlap, an illegal operation in SimpleITK. The later type of entry denoting potential errors is used to relax the requirement for an external viewer. A cell using the SimpleITK Show function which relies on an external viewer may or may not generate an error depending on whether the viewer is installed or not, such as on our continuous integration testing service machines. All output cells are checked to ensure that the expected errors were generated and that no unexpected errors occurred. Potential errors, if generated, are ignored.

#### Dependencies

As the notebook development environment also depends on additional packages, we readily facilitate their installation.

For the R programming language, all of the dependencies are readily installed from the R prompt using a list we provide.

In Python, we support two setups, plain Python, and Anaconda Python. For both options, we recommend the use of Python virtual environments. These enable one to maintain a consistent and reproducible development environment with known versions of each package. When using plain Python, the user first creates a virtual environment and then installs the dependencies using the pip package management system and the requirements file we provide. From the command line, issuing the command pip install -r requirements.txt is all that is required. When using the Anaconda Python distribution, the virtual environment and dependent package installation are created using the conda package management system. From the command line, issuing the command conda env create -f environment.yml will create a virtual environment called “sitkpy” which contains all the required packages.

#### Data Sharing

Finally, the last component in our development environment infrastructure addresses the need for convenient data sharing and distribution. The development environment does not contain the data referred to in the notebooks. Rather, the data is stored in multiple instances of the MIDAS data storage system (www.midasplatform.org) and on plain websites such as the Point-validated Pixel-based Breathing Thorax Model (POPI) [26]. We use a data directory which serves as a local cache of all the datasets. Initially, the cache only contains a data manifest. This file is a JSON document containing a list of file names and their MD5 hash function values and an optional URL. The user fetches a specific dataset by using the fetch_data method which we provide. The input to this method is the file name as specified in the data manifest. If the file is already in the local data cache, then no download is performed. If the file is not in the cache and a URL was specified in the data manifest file, then we attempt to download it from the URL and cache it locally. If no URL is specified, then we attempt to download it from the list of MIDAS repositories we maintain. The MIDAS system allows one to download data based on its MD5 hash value which uniquely identifies it. Once the data is found on one of the repositories, it is downloaded. After download either from MIDAS or directly from a URL, the MD5 hash value for the downloaded file is automatically computed and compared to the value specified in the data manifest file. If the two match, then the download is successful and the data is valid; otherwise, an error is issued.

This data sharing approach allows anyone using the development environment to readily share data stored online with multiple collaborators in a simple and convenient manner. The only information that needs to be directly shared is the data manifest file.

## Results

The SimpleITK Image-Analysis Notebook environment and an extensive set of more than 35 notebooks is freely available on GitHub under an Apache 2.0 license. The repository can be found under the Insight Software Consortium organization: github.com/InsightSoftwareConsortium/SimpleITK-Notebooks.

The notebooks found in the repository include material which is useful for educational purposes and for illustrating the ability of the environment to serve as a platform for reproducible research. Note that this work is in line with the current scientific trend towards development of free, open-source, computing environments such as those offered by the Python and R programming languages.

We next present case studies illustrating notebooks for educational purposes and for reproducible research. In each category, we describe both a Python-based notebook and an R notebook.

### Educational Notebooks

The educational notebooks introduce concepts associated with incorporating SimpleITK into contemporary problem settings. We provide notebooks illustrating the usage, interaction, and modification of images and transforms in the two most prevalent research development languages, Python and in R. These notebooks focus on illustrating common SimpleITK usage paradigms, rather than developing new research algorithms.

The SimpleITK and Jupyter notebook environment is well suited to teach the tool and techniques need to incorporate multi-dimensional image processing into medical, biological, and industrial research endeavors. Special emphasis is given to using these tools to address big data challenges associated with volume, velocity, veracity, and variety of real-world imaging data. The Jupyter notebooks allow students that may have differing levels of programming expertise to uniformly experience developing and applying tools in contemporary problem settings with motivating problem domains that include object detection, scene segmentation, and object co-localization (i.e., registration). The paradigms demonstrated employ best practices needed for generating reproducible results by including version management for both the development and analysis phases of a research project. Students are exposed to a rich set of tools: high performance multi-threaded image processing algorithms (exposing ITK), rapid prototyping (Jupyter notebooks), and analysis pipeline building, Statistical Analysis (R), machine learning tools (R, scikit-learn), and reproducible science tools (R, python, git).

#### Transformations

The first educational notebook we describe here provides an overview of the transformation types supported by SimpleITK and illustrates how to move from a low-dimensional parameter space to higher ones, starting with a rotation or translation going through rigid and similarity transformations to affine. We also emphasize the non-standard parameterization associated with the global transformations. All global transformations except translation are centered. That is, they are of the following form:

$$T(\mathbf{x}) = A(\mathbf{x}-\mathbf{c}) + \mathbf{t} + \mathbf{c} $$

**Fig. 5:**
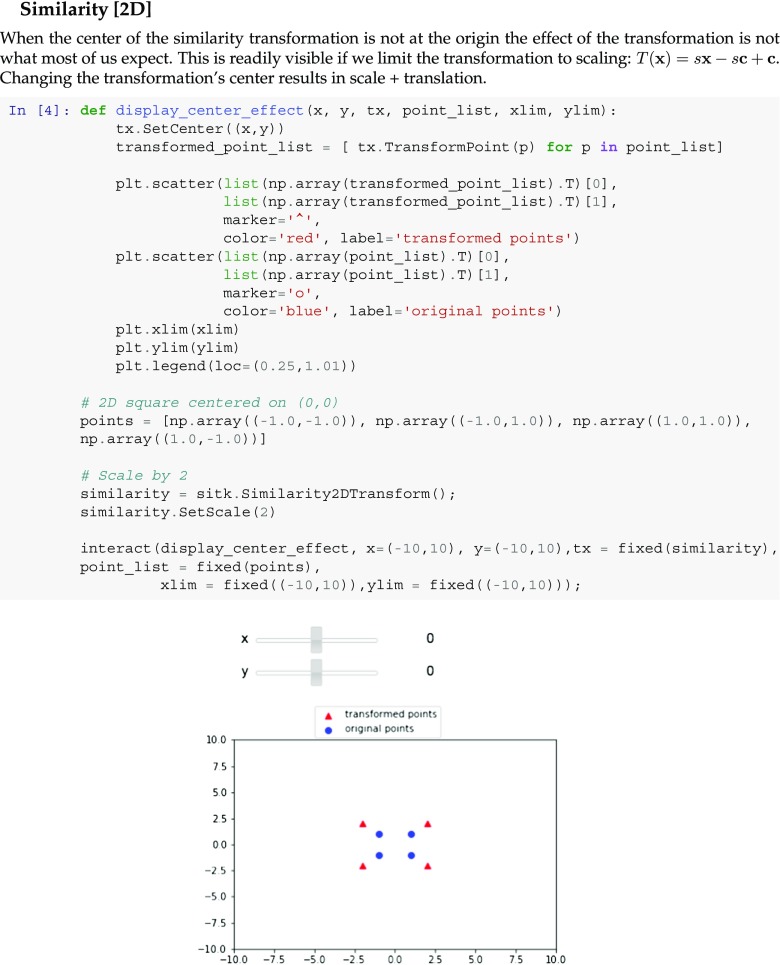
Snippet from a Python educational notebook illustrating the concepts underlying the SimpleITK transformation model. This section allows the user to interactively change the transformation center using sliders and observe its somewhat unintuitive effects on a similarity transformation. Changing the center also results in translation. A similar notebook is available in R

The nomenclature describing this transformation refers to the following components: matrix *A*, center **c**, translation **t**, and offset **t** + **c** − *A* **c**. The use of centered transformations may sometimes surprise the newcomer as we illustrate in the notebook snippet shown in Fig. 5.

Additionally, this notebook introduces the two types of bounded domain transformations supported by SimpleITK, BSpline Free-Form Deformations and Displacement Fields. The notebook illustrates the details associated with the creation of each of these transformation types and with the composite transform which was described above. The notebook concludes with code illustrating the SimpleITK approach to reading and writing transformations.

#### Image Operations

**Fig. 6:**
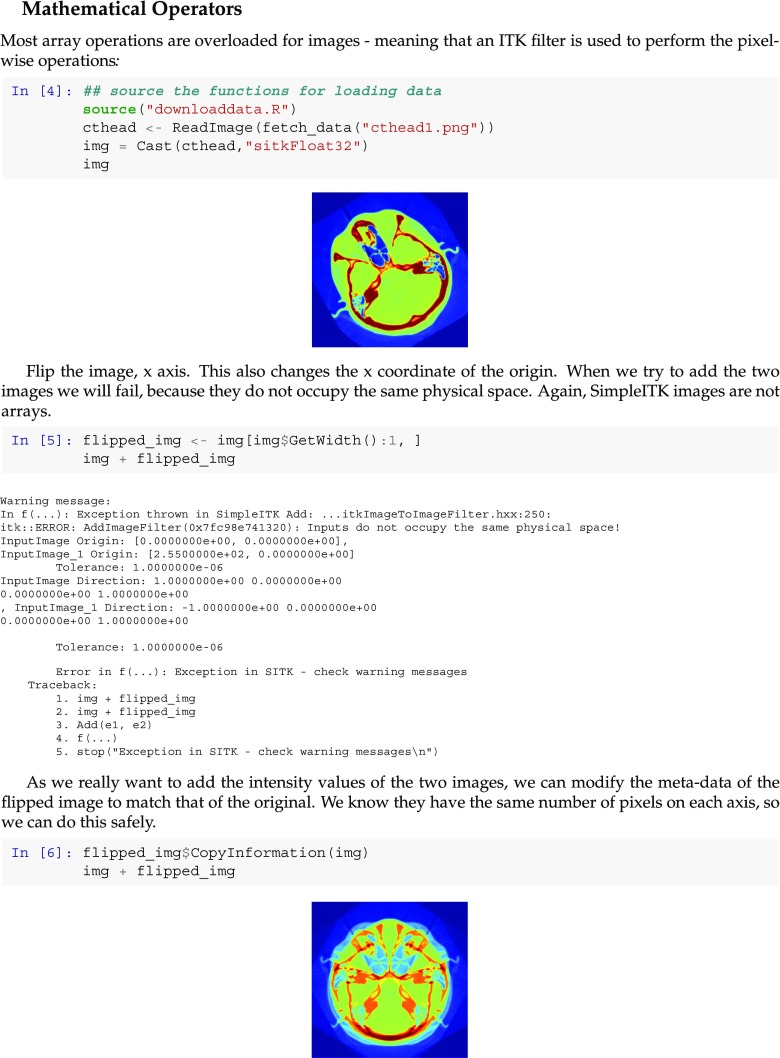
Snippet from an R educational notebook illustrating the concepts underlying the SimpleITK image model. This section explains the conceptual difference between an image and an array. In SimpleITK binary operations, such as adding intensities of two images, are only valid if the images occupy the same physical space. A similar notebook is available in Python

The second educational notebook we describe provides an overview of image access operators (slicing and indexing), mathematical operators between images and logical comparison operators. The code illustrates the tight integration of SimpleITK with the specific programming languages, providing familiar access methods with the language specific indexing conventions. Additionally, we illustrate what happens when one forgets SimpleITK’s fundamental tenet about images, all of these operations are only valid if the images occupy the same physical location in space. We also show how to explicitly coerce two images to occupy the same space, so that a developer who insists on treating images as arrays can do so, but they have to do so explicitly. The notebook snippet shown in Fig. 6 illustrates this.

### Reproducible Research Notebooks

A set of reproducible research notebooks based on published research is also available. More specifically, variations on the work described in [22], metrics for evaluation of segmentation, and [29] spherical fiducial localization in cone-beam CT.

#### Segmentation Evaluation

In our first notebook example, we partially reproduce the work described in [22], evaluating liver tumor segmentations. While the focus of SimpleITK is on segmentation and registration algorithms, the toolkit also provides components that facilitate algorithm validation and evaluation. The main requirements on validation listed in [12] include standardization of validation methodology, use of reference data sets and use of appropriate validation metrics. The work presented here addresses the later two.

Similar to Popa et al., we start by deriving a reference segmentation from multiple manual expert segmentations. As the authors publicly provided the set of liver tumor segmentations used in their work, we utilize that data. We illustrate the two methods supported by SimpleITK for determining a reference segmentation from multiple segmentations, majority vote, and the STAPLE algorithm [27]. Once we have a reference segmentation, we compare segmentations to it. Similar to the original work, we use both volumetric overlap measures and surface distance measures between the two segmentations. The former are directly computed using SimpleITK filters. The later require a slightly more involved computation.

**Fig. 7:**
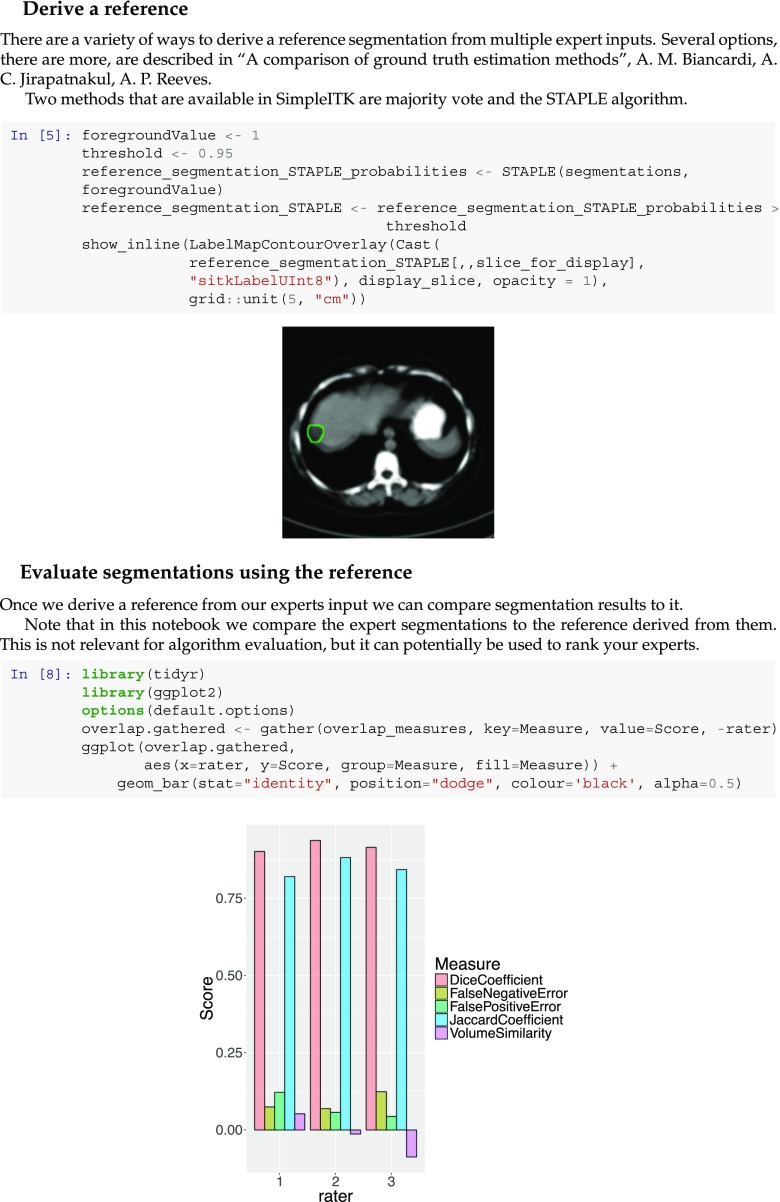
Snippet from an R notebook reproducing work from [22]. The code shows derivation of a reference segmentation using the STAPLE algorithm and plotting of volumetric overlap measures. A similar notebook is available in Python

To compute the non-symmetric distance measures between the two surfaces defined by the reference and evaluated segmentation, we compute a distance map for the reference segmentation. We then extract the surface of the evaluated segmentation and use these two elements as input for the LabelIntensityStatisticsImage Filter. This filter computes statistics on the intensities in the spatial region defined by a label mask. In our case, the intensities correspond to the distance from the reference segmentation surface and the label mask corresponds to the evaluated segmentation’s surface, yielding information on the distances between the two surfaces. In the notebook environment, these numbers are readily plotted using either the ggplot2 package [28] in R or the matplotlib package [11] in Python. The notebook snippet shown in Fig. 7 illustrates the derivation of a reference segmentation and plotting of the evaluation results.

#### Fiducial Localization

In our second notebook example, we partially reproduce the work described in [29], spherical fiducial localization in cone-beam CT. We implement the method used to establish a reference localization of the markers in the volumetric data. In addition to the edge-based localization used in the original work, we also present an intensity-based localization approach using the Otsu threshold selection method.

**Fig. 8:**
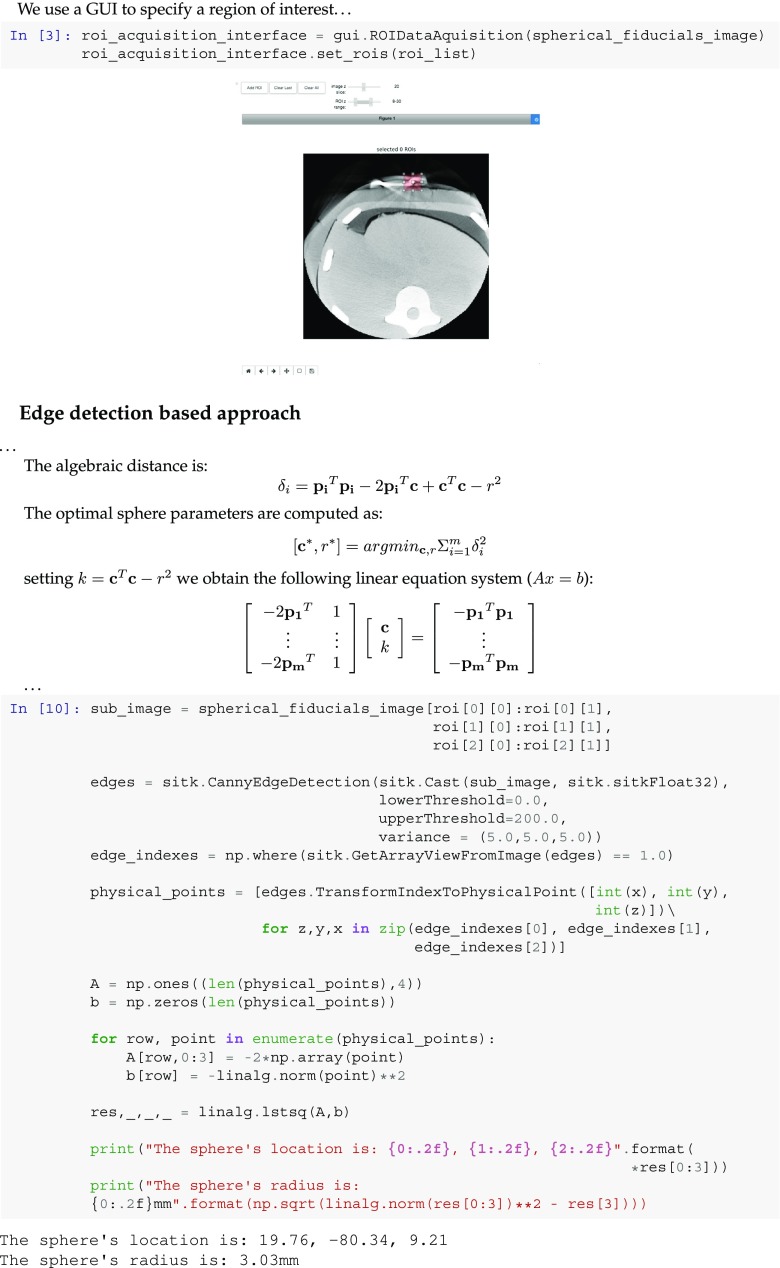
Snippet from a Python notebook reproducing work from [29]. Code shows GUI for selecting a region of interest (ROI) which bounds a spherical fiducial. The fiducial is then localized via least-squares fit of a sphere to the edges detected in the ROI. A similar notebook is available in R

Both approaches start with the user interactively selecting a box-shaped region of interest which bounds the fiducial, using the ROIDataAquisition GUI component. With the edge-based approach, we localize the edges in the ROI using the Canny edge detector and then estimate the optimal sphere which fits the edges using a least-squares formulation. The notebook snippet shown in Fig. 8 describes this formulation and its implementation using SimpleITK and the SciPy library [14]. With the thresholding-based approach, once the fiducial is segmented we use SimpleITK’s LabelShapeStatisticsImage Filter component to obtain its location as the centroid of the segmented blob and its radius as the radius of the sphere which has the same volume as the segmented blob.

## Discussion and Conclusions

Two significant requirements associated with modern scientific computational environments are that they facilitate collaborations and that they enable reproducibility [1, 2, 7, 21]. In this work, we presented such an environment for developing biomedical image analysis workflows using a literate programming approach with the Python or R programming languages. The environment allows one to provide algorithmic details, mathematical formulations, figures, and implementation code all within a single document. Data sharing is readily supported via remote online data repositories and raw URL addresses. Additionally, the environment supports continuous integration testing of notebooks, further facilitating collaborations, as all contributions to a repository are tested before merging into it. We focused our work on the Python and R interpreted programing languages from among those supported by SimpleITK due to their popularity within the scientific computing community, though developing SimpleITK notebooks using other languages is possible as long as there is a Jupyter Kernel which supports the language.

The need for an environment that facilitates the development of image-analysis workflows for non-expert C+ +developers which also incorporates the algorithms found in ITK is not new. ITK components have been previously incorporated into a variety of image-analysis environments. The main challenge facing developers incorporating ITK components is in hiding the complexity underlying the C+ + code. As a consequence, the majority of solutions are either stand-alone programs that provide a graphical interface to various ITK functionalities or development environments based on a visual programming methodology in which analysis pipelines are constructed by connecting and interacting with graphical representations of ITK components. Examples of standalone programs include 3D Slicer [8], ITK-SNAP [30] and the MITK Workbench [19]. Examples of visual programming environments include SCIRun [20], MeVisLab [23], ITKBoard [17], and SimITK [6]. The only two efforts, of which we are aware, that incorporate ITK components directly into an interpreted programming environment include [5], incorporating ITK into the MATLAB scripting language (MathWorks, Natick, MA, USA), and the effort of incorporating ITK into the Python language initiated by the ITK developers [13]. The former effort appears to have been abandoned. The latter is active with ITK Python bindings available for all major platforms. This effort is the closest to SimpleITK with the primary differences being that it is focused only on Python and that it does not provide the simplified interfaces available in SimpleITK.

The use of standalone programs to perform complex image-analysis tasks often results in computational workflows that are hard to document and replicate. This may also lead to disconnected workflows if no single program supports all of the required functionality without modification. Visual programming environments are more flexible and reproducible in that they allow the user to specify and save the analysis pipeline with a relatively fine-grained control over the ITK components. The only visual programming environment listed above which also allows one to incorporate a rich set of components developed by other users is SimITK which fits within MATLAB’s Simulink environment. Thus, in most cases, extending the visual programming environment is beyond the user’s expertise.

In this work, we leveraged the Jupyter notebook web application in conjunction with the SimpleITK toolkit to offer a flexible and extensible development environment with a focus on facilitating both educational and reproducible research workflows. Beyond the advantages of this environment described above, the use of the popular Python and R programming languages reduces the amount of effort required to master the syntax and semantics of the environment. Additionally, the large number of packages freely available for these programing languages are readily integrated into the environment, easily extending it based on the developer’s needs.

To download, contribute, or inquire on the SimpleITK Image-Analysis Notebook environment go to: github.com/InsightSoftwareConsortium/SimpleITK-Notebooks.

## References

[1] Adams J (2012). Collaborations: the rise of research networks. Nature.

[2] Adams J (2013). Collaborations: the fourth age of research. Nature.

[3] Avants BB, Tustison NJ, Stauffer M, Song G, Wu B, Gee JC (2014). The insight toolkit image registration framework. Front Neuroinform.

[4] Chacon S (2009). Pro Git.

[5] Chu V, Hamarneh G: MATLAB-ITK interface for medical image filtering, segmentation, and registration. In: SPIE Medical imaging: image processing, vol 6144, 2006

[6] Dickinson AWL, Abolmaesumi P, Gobbi DG, Mousavi P (2014). SimITK: visual programming of the itk image-processing library within simulink. J Digit Imaging.

[7] Donoho DL, Maleki A, Rahman IU, Shahram M, Stodden V (2009). Reproducible research in computational harmonic analysis. IEEE Comput Sci Eng.

[8] Fedorov A, Beichel R, Kalpathy-cramer J (2012). 3D Slicer as an image computing platform for the quantitative imaging network. Magn Reson Imaging.

[9] Gamma E, Helm R, Johnson R, Vlissides JM (1994). Design patterns: elements of reusable object-Oriented software.

[10] Hermans F (2016). Leaders of tomorrow on the future of software engineering: A roundtable. IEEE Softw.

[11] Hunter JD (2007). Matplotlib: a 2D graphics environment. Computing In Science & Engineering.

[12] Jannin P, Fitzpatrick JM, Hawkes DJ, Pennec X, Shahidi R, Vannier MW (2002). Validation of medical image processing in image-guided therapy. IEEE Trans Med Imaging.

[13] Johnson HJ, McCormick M, Ibáñez L (2015). The ITK software guide: design and functionality.

[14] Jones E, Oliphant T, Peterson P. et al (2017) SciPy: open source scientific tools for python

[15] Kluyver T, Ragan-Kelley B, Pérez F, Granger B, Bussonnier M, Frederic J, Kelley K, Hamrick J, Grout J, Corlay S, Ivanov P, Avila D, Abdalla S, Willing C, Team JD: Jupyter notebooks - a publishing format for reproducible computational workflows. In: Positioning and power in academic publishing: players, agents and Agendas: proceedings of the 20th international conference on electronic publishing, 2016, pp 87–90

[16] Knuth DE (1984). Literate programming. Comput J.

[17] Le HD, Li R, Ourselin S, Potter J: A visual dataflow language for image segmentation and registration. In: Software and data technologies: second international conference, ICSOFT/ENASE, 2009, pp 60–72

[18] Lowekamp BC, Chen DT, Ibáñez L, Blezek D (2013). The design of SimpleITK. Front Neuroinform.

[19] Nolden M, Zelzer S, Seitel A et al: The medical imaging interaction toolkit: challenges and advances 10 years of open-source development 8(4):607–620, 2013 10.1007/s11548-013-0840-8 23588509

[20] Parker SG, Johnson CR: SCIRun: a scientific programming environment for computational steering. In: Proceedings of the 1995 ACM/IEEE conference on supercomputing, 1995

[21] Peng RD (2011). Reproducible research in computational science. Science.

[22] Popa T, Ibáñez L, Levy E, White A, Bruno J, Cleary K: Tumor volume measurement and volume measurement comparison plug-ins for volview using itk. In: SPIE medical imaging: visualization, image-guided procedures, and display, 2006

[23] Ritter F, Boskamp T, Homeyer A, Laue H, Schwier M, Link F, Peitgen HO (2011). Medical image analysis: a visual approach. IEEE Pulse.

[24] Schindelin J, Arganda-Carreras I, Frise E (2012). Fiji: an open-source platform for biological-image analysis. Nat Meth.

[25] Shen H (2014). Interactive notebooks: sharing the code. Nature.

[26] Vandemeulebroucke J, Sarrut D, Clarysse P: The POPI-model, a point-validated pixel-based breathing thorax model. In: Proceeding of the XVth ICCR conference, 2007

[27] Warfield SK, Zou KH, Wells WM (2004). Simultaneous truth and performance level estimation (STAPLE),: an algorithm for the validation of image segmentation. IEEE Trans Med Imaging.

[28] Wickham H (2009). ggplot2: elegant graphics for data analysis.

[29] Yaniv Z (2009). Localizing spherical fiducials in C-arm, based cone-beam. CT Med Phys.

[30] Yushkevich PA, Piven J, Cody Hazlett H, Gimpel Smith R, Ho S, Gee JC, Gerig G (2006). User-guided 3D active contour segmentation of anatomical structures: significantly improved efficiency and reliability. Neuroimage.

